# Development of an in vitro tissue culture system for hammer coral (*Fimbriaphyllia ancora*) ovaries

**DOI:** 10.1038/s41598-021-03810-x

**Published:** 2021-12-21

**Authors:** Yi-Ling Chiu, Ching-Fong Chang, Shinya Shikina

**Affiliations:** 1 Atmosphere and Ocean Research Institute, The University of Tokyo, Chiba, Japan; 2 Center of Excellence for the Oceans, National Taiwan Ocean University, 2 Pei-Ning Rd., Keelung, 20224 Taiwan; 3 Institute of Marine Environment and Ecology, National Taiwan Ocean University, Keelung, Taiwan; 4 Department of Aquaculture, National Taiwan Ocean University, Keelung, Taiwan

**Keywords:** Biotechnology, Cell biology

## Abstract

In vitro gonad culture systems have proven useful to investigate intrinsic mechanisms of sexual reproduction in animals. Here we describe development of an in vitro culture method for coral ovaries. Mesenterial tissues containing both ovaries and mesenterial filaments were microscopically isolated from the scleractinian coral, *Fimbriaphyllia ancora*, and culture conditions were optimized. M199 diluted 10× (10% M199, pH 8.1) and supplemented with 25 mM HEPES and the antibiotics, ampicillin, penicillin and streptomycin, supported oocyte survival and maintained the structural integrity of ovaries during short-term culture (~ 6 days). Addition of a commercial antibiotic–antimycotic solution (Anti–Anti) and fetal bovine serum adversely affected ovary maintenance and caused tissue disintegration. Characterization of cultured ovaries showed that there is no difference in cell proliferation of ovarian somatic cells between culture Days 1 and 6. Moreover, the presence of oogonia and expression of a major yolk protein, vitellogenin, were confirmed in ovaries cultured for 6 days. This system will be useful for studying effects of a wide range of substances on coral oogenesis.

## Introduction

Sexual reproduction of scleractinian corals is a biological phenomenon that continues to fascinate researchers and the general public. Since the 1980s, many studies have investigated sexual reproduction in corals worldwide, resulting in improved understanding of environmental factors influencing gametogenesis and spawning/brooding^1–4^. Nonetheless, there remain many unanswered questions about intrinsic mechanisms controlling coral sexual reproduction.

Corals are marine organisms belonging to the phylum Cnidaria. Like other cnidarians (sea anemones, jellyfish, hydra, etc.), corals are diploblastic, having a relatively simple body structure. Complex organs corresponding to vertebrate brains, blood vessels, and intestines are lacking in corals^5,6^. Corals reproduce both asexually and sexually. For sexual reproduction, many corals develop gametes within polyps over a period of several months to a year and release them at specific times^7^. Coral germ cells generally develop within specific regions of mesenterial tissues in the polyp^7^. These regions are usually small swellings, conventionally called gonads. Coral gonads are composed of several cell types, including germ cells, gonadal somatic cells, and neurons^7,8^.

In many animals, germ cell proliferation, differentiation, and/or maturation are regulated by intrinsic factors such as neuropeptides, steroid hormones, and biogenic amines^9–13^. Information about corresponding compounds in corals is important to better understand both basic and applied biology. For example, identifying similarities and differences between corals and vertebrates could provide insight into evolution of sexual reproduction in metazoans^14^. Additionally, coral propagation projects are being undertaken worldwide to restore damaged coral reefs^15–19^. Asexual propagation is the method commonly used worldwide^19^. Identification of intrinsic factors regulating coral gametogenesis would make it possible to induce sexual reproduction artificially, which could lead to efficient production of genetically diverse coral seedlings.

Our recent transcriptome analysis of coral gonads showed that genes encoding growth factors, neuropeptides, and neurotransmitter receptors are expressed in the gonads^20^, leading us to hypothesize that coral gametogenesis is regulated by those intrinsic factors. To test this hypothesis, functional analysis of genes and proteins is essential. However, development of related techniques for corals has been slow. Although gene knockdown or knockout has recently been accomplished in *Acropora* species^21,22^, these techniques are only applicable during the embryonic stage.

In vitro tissue and organ culture systems are powerful tools for investigating intrinsic mechanisms of sexual reproduction. For example, in fish, the mechanism of hormonal regulation during gametogenesis has been investigated by adding steroids or growth factors to the culture systems and observing responses of germ cells at the molecular and cellular levels^23–25^. Establishment of a similar in vitro culture system for coral gonads should provide a useful platform to study effects of a wide range of substances on coral gametogenesis.

There have been no reports of in vitro culture techniques for coral gonads. Therefore, we developed a technique to culture ovaries in vitro for short periods while maintaining oocyte survival and their three-dimensional structure. A coral species, *Fimbriaphyllia ancora*, which is widely distributed in the Indo-Pacific Ocean, was used. This species is gonochoric (separate male and female organisms) and has large polyps (3–5 cm in diameter). Moreover, a technique for isolating mesenterial tissues encompassing ovaries is well established^26^. Furthermore, antibodies identifying germ cells or yolk proteins in the ovaries are available for this species^26,27^. We first optimized culture conditions by assessing effects of antibiotics/antifungal agents, types of culture medium, medium pH, and fetal bovine serum on oocytes, using histological analysis. We then characterized ovarian cells cultured under optimized conditions.

## Materials and methods

### Sampling of experimental animals

Three large female colonies of *F. ancora* were selected and labeled at Nanwan Bay in southern Taiwan (21°57′N, 120°46′E). In March and April 2016, before the spawning season in May, scuba divers collected coral segments (~ 5–6 cm in length) from each labeled colony with approval from the Kenting National Park administration. Collected fragments were subsequently transferred to an aquarium at National Taiwan Ocean University (NTOU). Dissection of the coral and gonad isolation were carried out in accordance with guidelines for Institutional Animal Care and Use from NTOU.

### Isolation and observation of mesenterial tissues

Gonads and associated mesenterial filaments (mesenterial tissue) were microscopically isolated from individual polyps and pooled in a culture dish. In order to minimize mechanical damage, ovaries were not separated from mesenterial filaments. Isolated mesenterial tissues were washed three times with filtered sterile seawater (FSW, sterile, pH 8.1) supplemented with antibiotics (50 µg/mL ampicillin, 50 U/mL penicillin and 50 µg/mL streptomycin) to inhibit bacterial growth. All procedures were performed in a laminar flow hood. Observations employed a stereomicroscope (SZX16; Olympus) or a fluorescence microscope (IX71SF1, Olympus). Endogenous red fluorescent protein (RFP) of oocytes was used as an oocyte marker and observed with a U-MWIG 2 filter (excitation wavelength 520–550 nm, emission wavelength 580 nm) on a fluorescence microscope^28^.

To examine effects of antibiotics and antifungals, 50 µg/mL ampicillin, 50 U/mL penicillin, 50 µg/mL streptomycin, or Antibiotic–Antimycotic (Anti–Anti, containing 100 units/mL of penicillin, 100 µg/mL of streptomycin, and 0.25 µg/mL of Amphotericin B, Thermo Fisher scientific, Waltham, MA) were selected, according to previous studies^29,30^. They were added individually, or in combinations of three antibiotics, or all together, and isolated mesenterial tissues were maintained under each condition for 6 days. As a control, tissues were maintained in FSW containing no antibiotics (Table 1).

**Table 1:** Antibiotics and antifungal tested in this study.

Group	Antibiotics/antifungal			
	Ampicillin	Streptomycin	Penicillin	Antibiotic–Antimycotic
Control	−	−	−	−
Treatment 1	+	−	−	−
Treatment 2	−	+	−	−
Treatment 3	−	−	+	−
Treatment 4	−	−	−	+
Treatment 5	+	+	+	−
Treatment 6	+	+	+	+

−: Not added to the cultures, +: Added to the cultures.

To investigate effects of culture medium, 4 commercially available culture media, Dulbecco’s modified Eagle’s medium (DMEM), Medium 199 (M199), RPMI 1640 medium (RPMI), Leibovitz’s L-15 medium (L-15) (all purchased from Thermo Fisher Scientific) were tested as used previously^29,30^. These media were diluted 10× with FSW^29^, and supplemented with 25 mM HEPES and antibiotics (50 µg/mL ampicillin, 50 U/mL penicillin, and 50 µg/mL streptomycin). To investigate effects of medium pH, 10% M199 (diluted with FSW, containing 25 mM HEPES and antibiotics) was adjusted to pH 7.8, 8.1, or 8.4. Effects of fetal bovine serum (FBS, Thermo Fisher Scientific) were also investigated and isolated tissues were cultured in 10% M199 (diluted with FSW, pH 8.1, containing 25 mM HEPES and antibiotics) supplemented with 1, 5, or 10% FBS. For each experiment, 3–4 isolated mesenterial tissues were cultured for 6 days in 3-cm laboratory dishes with 7.5 mL of culture media at 26 °C under standard atmospheric pressure with a 12-h light/dark cycle (light intensity: 17–19.5 μmol m^−2^ s^−1^). Culture media were replaced every 2 days. Cultures were observed and photographed under microscopes as described above at 0, 3, and 6 days of culture.

### Histological analysis

Samples were fixed with 20% Zinc Formal‐Fixx (Thermo Shandon, Pittsburgh, PA) for 16 h at room temperature, and preserved in 70% ethanol before use. Fixed samples were embedded in paraplast (Thermo Fisher Scientific), sectioned at a thickness of 4 µm, and stained with haematoxylin and eosin Y (HE, Thermo Fisher Scientific). Since approximately 600 serial sections comprise an entire isolated ovary, 10–30 sections were randomly photographed for each ovary, and used for subsequent analyses. To evaluate ovarian status, we developed two indicators, ovarian integrity and oocyte abnormality. Ovarian integrity is the ratio of ovarian area occupied by oocytes. This value (%) was estimated by measuring the total ovarian area and the area occupied by oocytes (oocyte area) on the ovarian section. Integrity of freshly isolated ovaries was 50–60% and this value decreased as oocytes disappeared from the ovary by cell death. Oocyte abnormality is the ratio of oocytes exhibiting morphological abnormalities (disintegrating cell membranes, appearance of unidentified vesicles, severe cytoplasmic deviations) in all oocytes of an ovary. The value (%) was determined by direct observation of histological sections. Three ovaries from three different colonies (9 ovaries in total) were analyzed. The total ovarian area and the area occupied by oocytes (oocyte area) in ovarian sections were determined by manual area measurements using ImageJ software (National Institutes of Health, Bethesda, MD).

### EdU assay

5-Ethynyl-2′-deoxyuridine (EdU, 50 µM) was added to the cultures at day 0 or day 5 of culture for 24 h. This labeled proliferating cells during days 0–1 or days 5–6 of culture. After EdU incubation, mesenterial tissues were washed three times with FSW supplemented with antibiotics (50 µg/mL ampicillin, 50 U/mL penicillin and 50 µg/mL streptomycin) and replaced with new culture media. Sample fixation and preparation of paraffin sections were performed as described in the section above (see “Histological analysis”). A Click-iT EdU Colorimetric IHC Detection Kit (C10644: Invitrogen, OR) was used to visualize proliferating cells. Eight to ten sections were randomly selected from each ovary, and percentages of ovarian somatic cells labeled with EdU were determined. Three ovaries from three different colonies (9 ovaries in total) were analyzed.

### RNA extraction, cDNA synthesis, and quantitative reverse transcription PCR

Ovaries were isolated from mesenterial tissues, and total RNA was extracted with TRIzol reagent (Thermo Fisher Scientific) following the manufacturer's protocol. First‐strand cDNA was synthesized from 1 μg of DNase‐treated RNA using SuperScript III reverse transcriptase (Thermo Fisher Scientific). Transcript levels of a major yolk protein, vitellogenin (Vg, GenBank accession no. KC777188), were determined by quantitative real‐time RT‐PCR, and compared between uncultured and cultured ovaries. *F. ancora* β‐actin (GenBank accession no. JQ968408) was used as a reference gene. Primers (Table 2) were designed using Primer Express 3.0 software (Applied Biosystems, USA) and primer efficiency was determined with a fivefold dilution series of the template cDNA. Only primer pairs that worked at 90–100% efficiency were used for quantitative PCR analysis. PCR was performed using a Bio‐Rad CFX Connect Real‐Time PCR detection system (Bio‐Rad Laboratories, Hercules, CA) with iQTM SYBR Green Supermix (Bio‐Rad Laboratories). Two‐step RT‐PCR was performed using the following conditions: (a) 1 cycle of 95 °C for 5 min, and (b) 40 cycles of 95 °C for 15 s and 60 °C for 1 min. Calculations were performed using the 2^−△△Ct^ method^31^. Reactions in which the template was omitted were used as negative controls for each primer set.

**Table 2:** List of the primers for quantitative PCR analysis.

Primer ID	5′–3′ sequence	Tm (°C)	Amplicon size (bp)
Vg Fw	GGAAATCACGGACCATCTCT	60	152
Vg Rv	GCTGGAATCTACTCTGCTTGC		
β-actin Fw	CGCCTTCCTTGGAATGGAATCCTCT	60	151
β-actin Rv	CTGCATCCTGTCAGCGATTCCAGG		

### Immunohistochemical analysis

Fixation of samples and preparation of paraffin sections were carried out as described in the section above (see Histological analysis). Immunohistochemistry was performed according to our previous methodology^32^. For primary antibody reactions, sections were incubated with affinity-purified anti-*F. ancora* piwi antibody (a marker for early stage germ cells, 1:4000^27^) or anti-*F. ancora* vitellogenin (Vg) antibody (1:4000^26^). For secondary antibody reactions, sections were incubated with a biotinylated goat anti-guinea pig IgG antibody (1:4000, Vector Laboratories, Burlingame, CA). Immunoreactive signals were visualized with 3,3′-diaminobenzidine (DAB; Sigma-Aldrich).

### Statistical analysis

Data are reported as means ± standard errors (SE). For comparisons of three or more groups, statistical significance was analyzed using Kruskal–Wallis tests (SPSS), followed by Dunn–Bonferroni pairwise comparisons. Statistical significance was analyzed with Mann–Whitney U-tests for comparisons between two groups. Statistical significance was set at *p* < 0.05.

## Results

### Morphological and histological characteristics of isolated mesenterial tissues in cultures and changes while maintained in seawater

**Figure 1:**
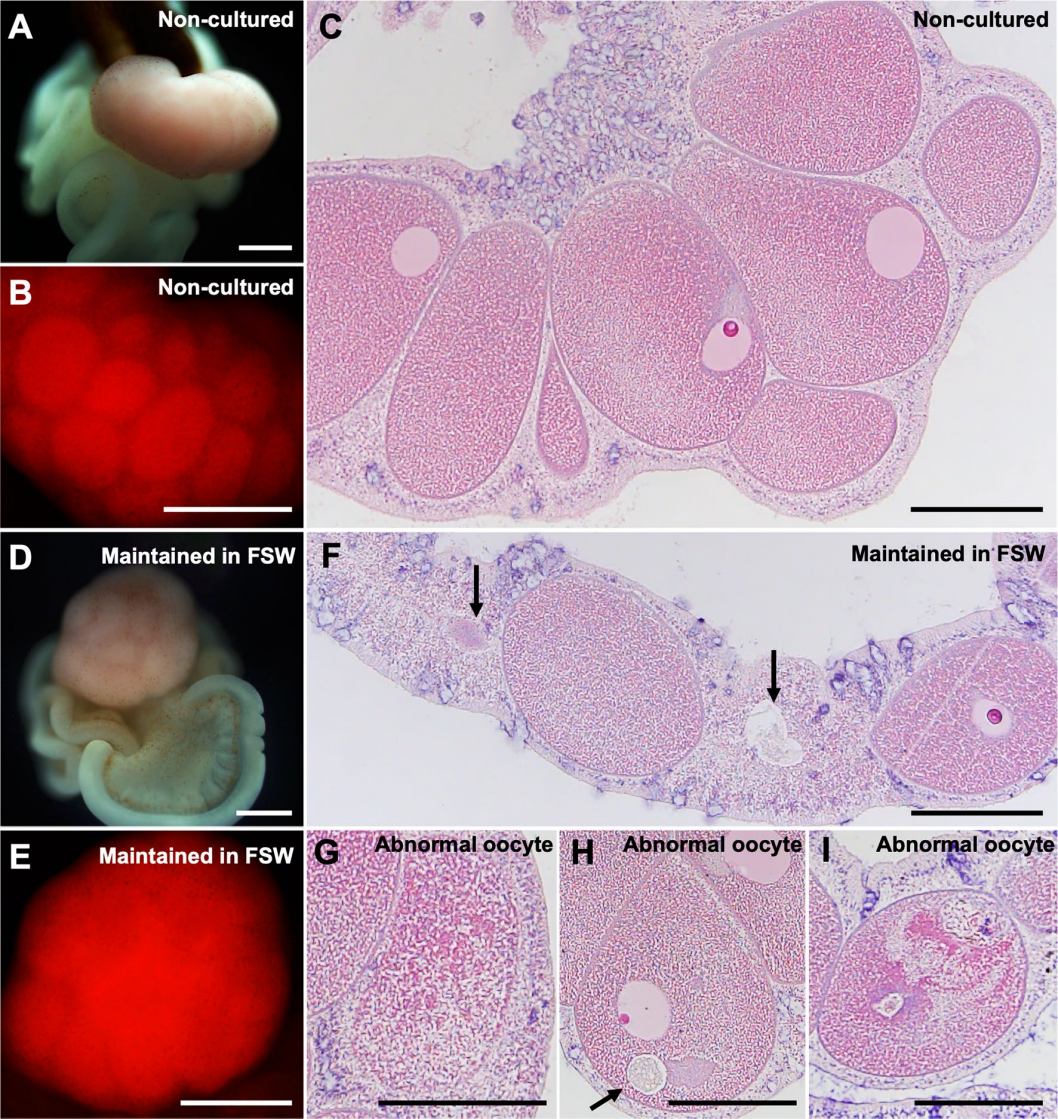
Morphological and histological characteristics of isolated mesenterial tissues and their changes during maintenance in FSW. (**A**) Brightfield view of freshly isolated mesenterial tissue containing an ovary and mesenterial filaments (Non-cultured). (**B**) Endogenous expression of oocyte RFP, observed with the U-MWIG 2 (RFP) filter of a fluorescence microscope. (**C**) HE section of the freshly isolated ovary (Non-cultured). (**D**) Brightfield view of mesenterial tissue maintained in FSW for 6 days. (**E**) U-MWIG 2 (RFP) filter view of an ovary maintained in FSW for 6 days. (**F**) HE section of an ovary maintained in FSW for 6 days. Oocyte disappearance was observed (arrows). (**G**–**I**) Morphological abnormalities in oocytes: disintegration of cell membranes (**G**), appearance of unidentified vesicles (**H**), and severe cytoplasmic deviations with unidentified vesicles (**I**). Scale bars = 500 μm (**A**,**B**,**D**,**E**,**F**); 200 μm (**C**,**F**,**G**,**H**,**I**).

Isolated mesenterial tissues, which include both ovaries and mesenterial filaments (Fig. 1A) were cultured. In culture, ovaries were generally observed as distinct swellings with pinkish color (Fig. 1A), and oocytes exhibited endogenous RFP^28^ (Fig. 1B). Mesenterial filaments exhibited slow movement, contracting and extending (Fig. 1B). Histologically, ovaries comprised oocytes with surrounding layers of ovarian somatic cells, and oocytes were filled with cytoplasm with evenly distributed small oil droplets (Fig. 1C).

When tissues were maintained in FSW for 6 days, contours of some oocytes, observed with RFP, became unclear (Fig. 1D,E). By contrast, no apparent changes were observed in morphological or behavioral characteristics of mesenterial filaments (Fig. 1D). Histological analysis of ovaries showed that some oocytes disappeared (Fig. 1F), or exhibited morphological abnormalities, such as disintegrating cell membranes (Fig. 1G), appearance of unidentified vesicles (Fig. 1H), or severe cytoplasmic aberrations with unidentified vesicles (Fig. 1I). Thus, oocytes degraded and/or underwent cell death during maintenance under FSW for 6 days. This raised the possibility that some nutrients essential for oocyte survival were missing in these cultures.

### Optimization of culture conditions

**Figure 2:**
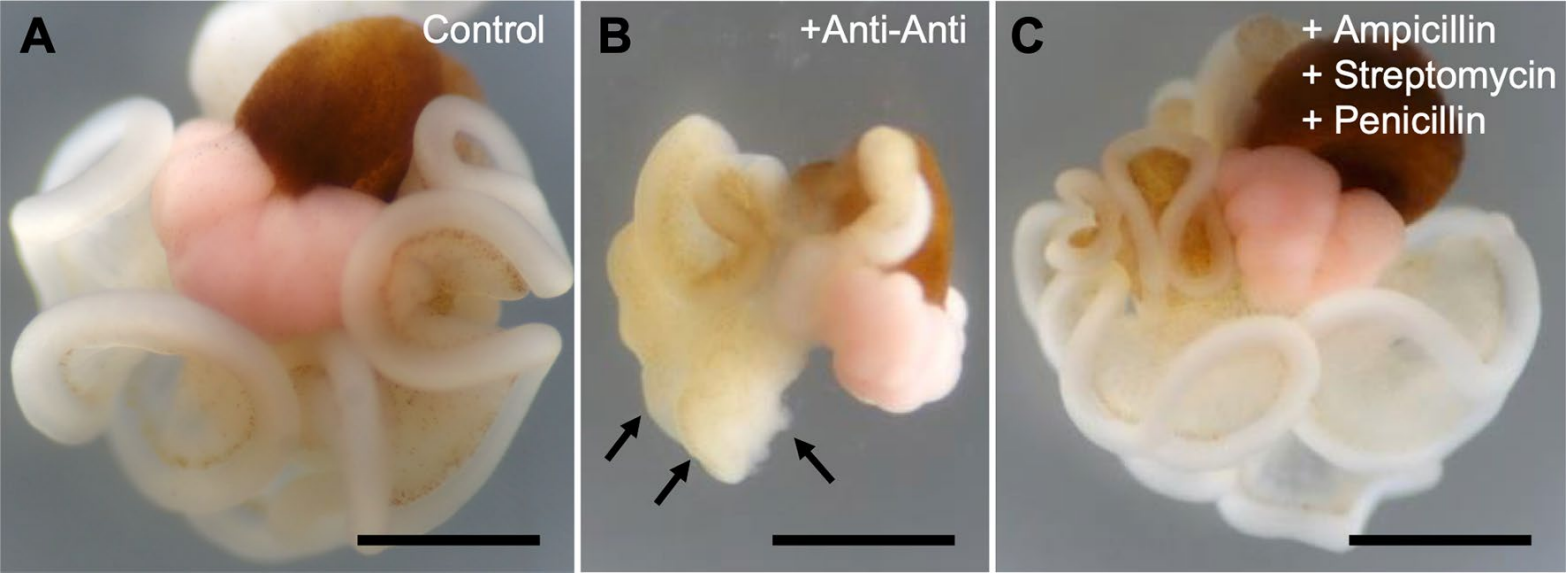
Effects of a commercially available antibiotic–antimycotic solution or antibiotics on mesenterial tissue. (**A**) Brightfield view of mesenterial tissue maintained in FSW for 3 days (Control). (**B**) Mesenterial tissue maintained in FSW supplemented with antibiotic–antimycotic solution (Anti–Anti, from Thermo Fisher Scientific) for 3 days. (**C**) Mesenterial tissue maintained in FSW supplemented with 50 μg/mL ampicillin, 50 U/mL penicillin and 50 μg/mL streptomycin for 3 days. Note that addition of Anti–Anti induced deformation of mesenterial tissues (arrows). Scale bars = 1 mm.

We therefore sought to optimize in vitro culture conditions that support oocyte survival. First, we attempted to identify suitable antibiotics and/or antifungal agents. Corals harbor a huge diversity of microorganisms (symbiotic algae, bacteria, etc.)^33^. Suppressing microbial growth is thus essential for any in vitro coral culture. Although addition of antibiotics and/or antifungal agents has been commonly used to reduce bacterial overgrowth, some of these additives induce disintegration of coral tissues^34,35^. Since disintegration of ovarian structure not only adversely affects oocyte survival, but is also incompatible with our objectives, we attempted to identify antibiotics and/or antifungal agents that permit maintenance of the structural integrity of whole mesenterial tissues. Addition of a commercial antibiotic–antimycotic solution (Anti–Anti) to the cultures (FSW), induced disintegration of mesenterial tissue (Fig. 2,B). In contrast, when three antibiotics (ampicillin, penicillin, and streptomycin) were added singly or in combination, little tissue disintegration was observed (Fig. 2C).

**Figure 3:**
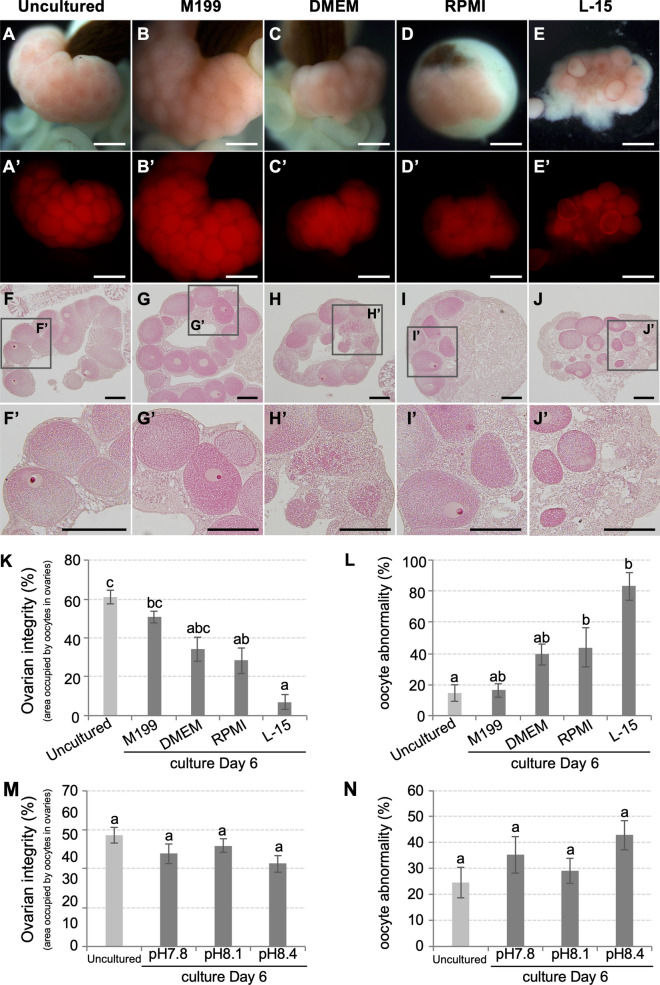
Effects of 4 commercially available culture media and medium pH. Brightfield views of freshly isolated mesenterial tissues (**A**) and mesenterial tissues cultured in M199 (**B**), DMEM (**C**), RPMI (**D**), or L-15 (**E**) for 6 days. (**A′**–**E′**) U-MWIG 2 (RFP) filter views of the same field as (**A**–**E**). HE sections of freshly isolated mesenterial tissue (**F**) and mesenterial tissues cultured in M199 (**G**), DMEM (**H**), RPMI (**I**), and L-15 (**J**) for 6 days. (**F′**–**J′**) Higher magnification views of the insets shown in (**F′**–**J′**). Effects of the 4 commercially available culture media (**K**,**L**) and medium pH (**M**,**N**) on ovarian integrity and oocyte status. Ovarian integrity was estimated by the ratio of the area occupied by oocytes in ovaries. Disappearance and abnormalities of oocytes in ovaries decrease this value. Oocyte status (oocyte abnormality) was examined histologically. Data are shown as means ± SE. Groups with different letters are significantly different (Kruskal–Wallis test, *p* < 0.05 and Dunn-Bonferroni post hoc test, *p* < 0.05). Scale bars = 500 μm (**A**–**E**,**A′**–**E′**); 200 μm (**F**–**J**,**F′**–**J′**).

Next, we investigated effects of 4 commercially available culture media (DMEM, M199, RPMI, and L-15) on *F. ancora* ovaries in the presence of the three antibiotics. To reduce microbial growth, all test media were diluted with FSW 1:10. HEPES (25 mM) was added to all media and the pH was maintained at 8.1. M199 effectively supports ovarian maintenance in culture. Microscopically, no differences were observed in ovarian appearance or oocytes, as indicated by RFP in uncultured (Fig. 3A,A′) and cultured ovaries in 10% M199 for 6 days (Fig. 3B,B′). In other groups, apparent disappearance of oocytes and disintegration of mesenterial tissues was observed in some samples (Fig. 3C–E,C′–E′). Histological analysis further demonstrated that ovaries cultured in 10% M199 had higher integrity, estimated by a higher ratio of area occupied by oocytes in ovaries, compared with those in other media (Kruskal–Wallis test, H = 29.148, df = 4, *p* < 0.05, Fig. 3F–J,F′–J′,K). Ratios of abnormal oocytes in ovaries cultured in 10% M199 were lower than those of other media (Kruskal–Wallis test, H = 22.577, df = 4, *p* < 0.05, Fig. 3F–J,F′–J′,L). No bacterial overgrowth was observed during the culture period. Based on this, 10% M199 was selected as the basic culture medium for *F. ancora* ovaries.

For further improvement of culture conditions, we next investigated effects of medium pH and addition of FBS on *F. ancora* ovaries. No significant differences were observed among ovaries cultured at pH 7.8, 8.1, or 8.4 (Ovarian integrity, Kruskal–Wallis test, H = 5.174, df = 3, *p* > 0.05, Fig. 3M and oocyte abnormality, Kruskal–Wallis test, H = 5.282, df = 3, *p* > 0.05, Fig. 3N). Addition of FBS (1–10%) adversely affected ovary maintenance in culture and caused disintegration of mesenterial tissue structure within 3 days (Fig. S1).

### Characterization of ovaries cultured under optimized conditions

**Figure 4:**
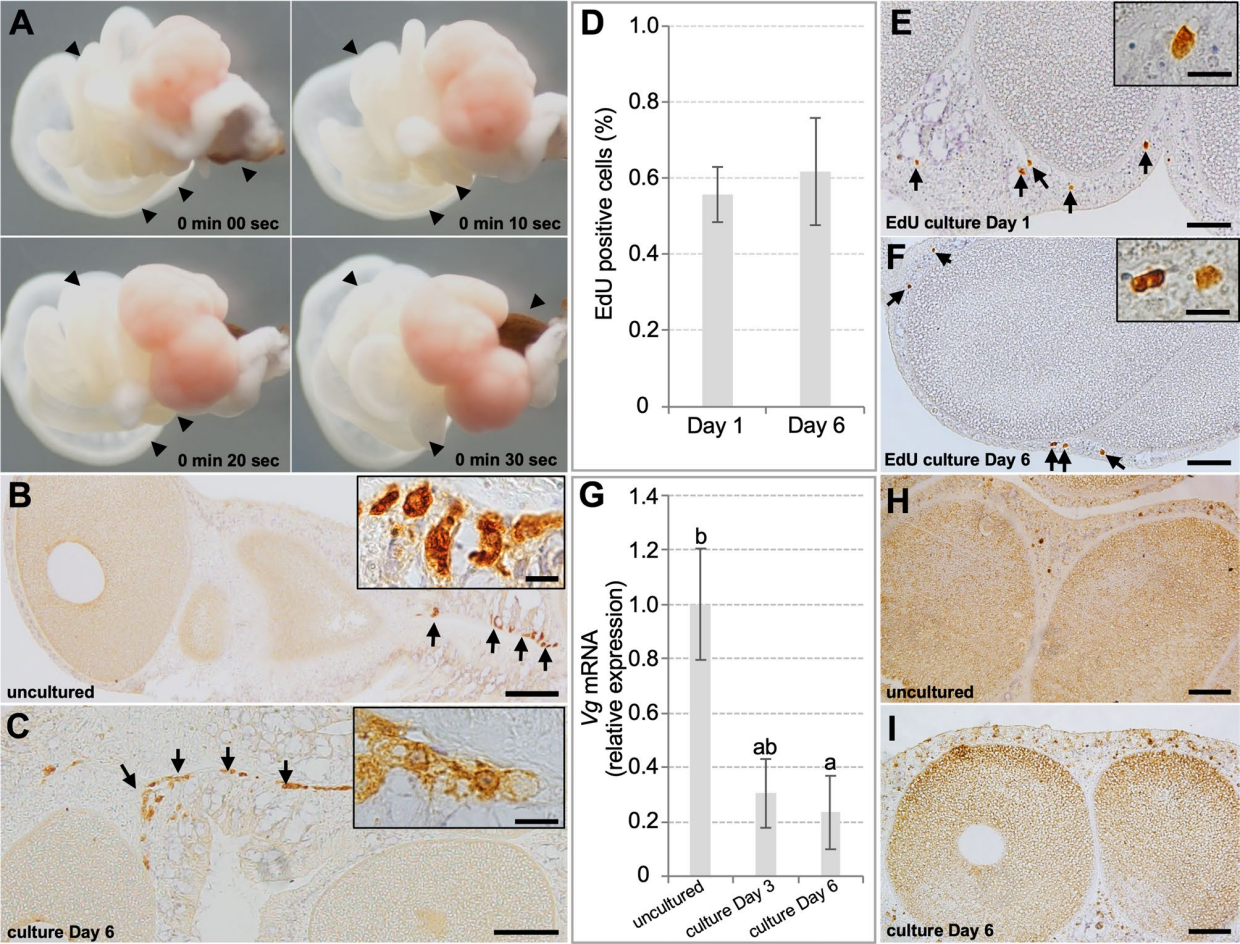
Status of ovaries cultured in optimized conditions. (**A**) Time-lapse images of mesenterial tissues (ovary and mesenterial filaments) cultured for 6 days in optimized conditions. The indicated mesenterial filaments (arrowheads) exhibited slow movements (see also Fig. S2). (**B**,**C**) Immunohistochemical detection of oogonia with anti-Eapiwi antibodies in an uncultured ovary (**B**) and an ovary cultured for 6 days (**C**). (**D**–**F**) Proliferating cells in cultured ovaries detected with an EdU assay. EdU was added to culture media (50 µM) at day 0 or day 5 of culture for 24 h. (**D**) The percentage of EdU-positive cells in ovarian somatic cells at day 1 and 6 of culture. Data are shown as means ± SE (n = 9 ovaries). EdU-positive cells (proliferating cells) detected in ovaries at culture Day 1 (**E**) or day 6 (**F**). Arrows indicate detected proliferating cells. (**G**–**I**) Expression of a major yolk protein, vitellogenin (Vg), in cultured ovaries. (**G**) Expression of Vg mRNA in uncultured ovaries and ovaries cultured in M199 for 3 days and 6 days. Data are shown as means ± SEs (n = 9) of relative values with those of uncultured ovaries. Groups with different letters are significantly different (*p* < 0.05). (**H**,**I**) Immunohistochemical detection of Vg in uncultured ovaries (**H**) and ovaries cultured for 6 days in M199 (**I**). Scale bars = 50 μm (**B**,**C**,**E**,**F**,**H**,**I**); 10 μm (insets in **B**,**C**,**E**,**F**).

Based on the above results, we then utilized 10% M199 (pH 8.1) supplemented with 25 mM HEPES and antibiotics (50 µg/mL ampicillin, 50 U/mL penicillin, 50 µg/mL streptomycin) as the culture medium for *F. ancora* ovaries. In this medium, structural integrity of ovaries and mesenterial filaments, as well as oocyte survival were well maintained at least for 6 days. Notably, mesenterial filaments continued to manifest slow movements (Fig. 4A and S2). Further characterization of cultured ovaries showed that oogonia were also maintained in cultured ovaries, as assessed by immunohistochemical detection (Fig. 4B,C). The EdU assay showed that there is no difference in cell proliferation activity of ovarian somatic cells between culture days 1 and 6 (Mann–Whitney, *p* > 0.05, Fig. 4D–F). mRNA expression of the yolk protein, vitellogenin (Vg), in cultured ovaries was also detected on culture days 3 and 6, although expression levels were lower than in uncultured ovaries (Kruskal–Wallis test, H = 8.856, df = 2, *p* < 0.05, Fig. 4G). Immunohistochemically, Vg protein was also detected in both somatic cells and oocytes of cultured ovaries, as in uncultured ovaries (Fig. 4H,I). These results indicate that coral ovaries can be cultured under our conditions for at least 6 days, maintaining ovarian structural integrity, proliferative activity of ovarian somatic cells, and some ovarian functions.

## Discussion

During the past four decades, progress has been made in culturing coral tissues/cells in vitro^36,37^. Although most coral tissues/cells have only been cultured as primary cultures without active growth, culture systems have been utilized to study coral-algal symbiosis^30,38^, biomineralization^34,39–43^, stress responses to heat^44^, and responses to marine pollutants^45^. Most recently, the first coral cell line was established from embryos of *Acropora tenuis*, and it is expected to be a very useful tool for comprehensive understanding of the coral organism and coral-algal symbiosis at the cellular and molecular levels^46^. Although the number of reports on coral tissue/cell culture is gradually increasing, to the best of our knowledge, there have been no published studies regarding in vitro culture of coral gonads/germline cells. The lack of research may reflect difficulties in sample preparation. Since most corals undergo sexual reproduction during a limited period every year^47–50^, this limits the time in which experiments can be carried out. Additionally, isolation techniques for live gonads have so far been reported only in *F. ancora*. In this study, despite the limited sampling period and the number of colonies we were permitted to sample, we successfully developed a culture system to enable oocyte survival and to maintain ovarian structure in culture for short periods. We also developed two indicators for assessing ovarian status, ovarian integrity and oocyte abnormality. Although the obtained data are still preliminary, and there is much room for improvement, the findings and techniques established in this study will facilitate studies of sexual reproduction in corals.

Culture media used for coral cell/tissue culture differ among species and target cell/tissue types. Conventionally, filtered sterile natural seawater (FSW), artificial seawater (ASW), or ASW supplemented with appropriate concentrations of commercial culture media (DMEM, L-15, M199, RPMI, etc.) have been used^29,30,37^. We found that 10% M199 improves maintenance of oocytes, as well as ovarian integrity in culture. M199 was first developed in 1950 for nutritional studies of chick embryo muscle fibroblasts^51,52^, and has been used in cell/tissue culture systems with a broad range of animals, including marine invertebrates such as sponges, corals, shrimp, abalones, and sea urchins^29,53–56^. M199 contains all 20 amino acids, and also more vitamins (ascorbic acid, vitamin A, vitamin D2) and additives (ATP, ADP, cholesterol, and guanine hydrochloride, etc.) than other media that were tested in this study. Vitamins are organic compounds that act as co-enzymes to augment catalytic activity^57^ and antioxidants^58^. Appropriate vitamin supplements improve cell viability, growth, and cell proliferation and differentiation of mammalian cells^59,60^. ATP comprises the primary means of energy storage and transfer in cells, driving a broad range of biological processes^61^. Exogenous ATP supplemented in the culture medium improves cell proliferation in rat astrocytes^62^ and has also been used to increase collagen and proteoglycan synthesis in chondrocyte tissue 3D culture^63^. At present, it is still unclear which components in M199 are responsible for improved performance of *F. ancora* ovarian cultures, because little is known about the nutrient and growth requirements of cnidarian tissue/cells culture^37^. It is probable that some amino acids, vitamins, or other additives in M199 medium solely or synergistically affect coral ovaries, and support survival and basal ovarian functions in culture.

FBS contains a variety of proteins, polypeptides, amino acids, glucose, growth factors, hormones, etc. that are essential for maintenance, attachment, growth and proliferation of cells^64^. FBS has been used in a broad range of cell/tissue cultures for both vertebrate and invertebrate cells^64^. In most studies of coral cell culture, FBS has been added to the mediums at a concentration of 5–20%^29,30^, and survival promoting effects were demonstrated in *Acropora microphthalma* endothelial cell culture^30^. However, we found that addition of FBS to culture media strongly induces disintegration of *F. ancora* ovaries. Since one of the objectives of this study was to maintain ovarian structure in vitro, we decided not to use FBS. Although it is currently unclear which factors in FBS cause this phenomenon, factors that inhibit intercellular adhesion of mesenterial tissues may be present.

The EdU assay demonstrated that proliferation of ovarian somatic cells was very low (~ 0.6%). This may be due to inherent characteristics of the ovaries at this stage. In a previous study, we reported a massive apoptotic phenomenon in gonadal somatic cells of *F. ancora*^8^. We found that while germ cells developed during gametogenesis, many gonadal somatic cells died by apoptosis, resulting in a significant decrease in the thickness and density of the gonadal somatic cell layer^8^. In the present study, ovaries were prepared a month or two before spawning. At this stage, active cell proliferation of gonadal somatic cells may not be occurring or may have been suppressed, as gonadal structure was being transformed to facilitate gamete release by reducing the number of somatic cells.

The mRNA level of yolk protein decreased in ovarian cultures from culture days 3–6. Although currently nothing is known about factors regulating yolk synthesis in corals, decreased expression levels are probably due to either the paucity of factors needed for maintenance of yolk synthesis or the existence of inhibitory factors in culture. Our previous study demonstrated that vitellogenin is produced by ovarian somatic cells^26^. Notably, in this study, no histological abnormality was observed in ovarian somatic cells, and cell proliferation appeared to be maintained during the culture period. These findings imply that our current culture conditions can at least support survival and proliferation of ovarian somatic cells but cannot fully support vitellogenin synthesis.

Although decreased expression of the *vitellogenin* gene was observed, it was indeed expressed in cultured ovaries. In future studies, vitellogenin expression may be used as an indicator to further improve culture conditions or to identify factors that promote yolk formation. For instance, by culturing ovaries under different temperatures, light intensities, wavelengths, pHs, etc., and by examining expression of vitellogenin, it may be possible to identify environmental factors that influence yolk formation. Similarly, it may also be possible to identify hormone-like substances that promote yolk formation, by culturing ovaries in the presence of sex steroids, neuropeptides, and monoamines, which, based on our previous transcriptomic and biochemical studies of *F. ancora*, are thought to exist^20,65,66^. Currently, the time that ovaries can be maintained in culture is limited to approximately 1 week, and in the case of prolonged cultivation, disappearance and/or morphological abnormalities of oocytes are observed after 9–10 days of culture. Nevertheless, because gene responses are usually detected within a few hours to a few days^67–69^, the established culture system could be used to identify those factors.

In summary, we have described the development of an in vitro tissue culture system for coral ovaries using the stony coral *F. ancora*. Our culture conditions support oocyte survival and structural integrity of ovaries for a short period (~ 6 days). This system will be a useful tool for investigating intrinsic mechanisms underlying coral oogenesis.

## Supplementary Information

Supplementary Legends.

Supplementary Figure S1.

Supplementary Figure S2.
