# Supplementary material for: Development of an in vitro tissue culture system for hammer coral (Fimbriaphyllia ancora) ovaries
Source: Sci Rep. 2021 Dec 21;11:24338. doi: 10.1038/s41598-021-03810-x (PMC8692509; doi:10.1038/s41598-021-03810-x)
Supplement: Supplementary file 1 — Supplementary Legends. [file 41598_2021_3810_MOESM1_ESM.docx]

**Supplementary Figure legends**

**Supplementary Figure S1.**

Effects of addition of fetal bovine serum (FBS) to mesenterial tissue. (**A**) Brightfield view of mesenterial tissue cultured in 10% M199, supplemented with 25 mM HEPES, antibiotics, and 1% FBS for 3 days. (**B**) U-MWIG 2 (RFP) filter views of the same field as in **A**. Mesenterial tissue exhibited disintegration.

**Supplementary Figure (video) S2.** Mesenterial tissue cultured for 6 days in 10% M199 (pH 8.1) supplemented with 25 mM HEPES and antibiotics. Mesenterial filaments exhibited slow movements.
