# Supplementary figures and images for: Development of an in vitro tissue culture system for hammer coral (Fimbriaphyllia ancora) ovaries
Source: Sci Rep. 2021 Dec 21;11:24338. doi: 10.1038/s41598-021-03810-x (PMC8692509; doi:10.1038/s41598-021-03810-x)

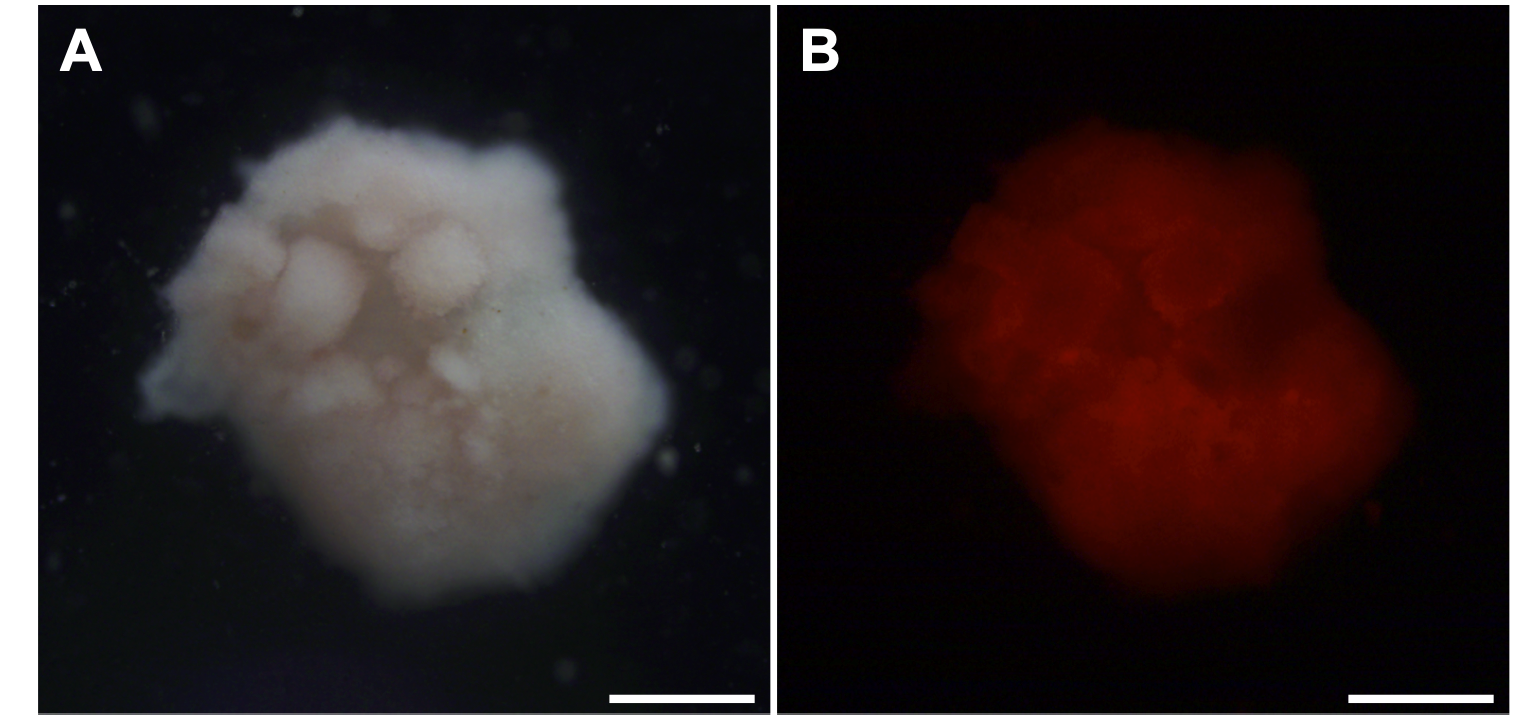

Supplement: Supplementary file 2 — Supplementary Figure S1. [file 41598_2021_3810_MOESM2_ESM.tiff]
